# Prevalence of Connective Tissue Disorder-Associated Interstitial Lung Disease Misdiagnosed and Treated As Tuberculosis

**DOI:** 10.7759/cureus.107678

**Published:** 2026-04-24

**Authors:** Gaurav Sahu, Tanushree Kothari, Nensi Singh, Shivani Chaturvedi, Shahzad Husain Arastu, Sharad Singour, Harsh Tripathi

**Affiliations:** 1 Pulmonary Medicine, LN Medical College and Research Center and JK Hospital, Bhopal, IND; 2 Internal Medicine, LN Medical College and Research Center and JK Hospital, Bhopal, IND

**Keywords:** autoimmune, connective tissue disease associated interstitial lung disease, india, interstitial lung disease, tuberculosis

## Abstract

Introduction: Connective tissue disease-associated interstitial lung disease (CTD-ILD) is an important contributor to the burden of diffuse parenchymal lung disease in India. In tuberculosis-endemic regions, overlapping clinical and radiological features frequently result in misdiagnosis as pulmonary tuberculosis and exposure to empirical anti-tubercular therapy (ATT), leading to delayed initiation of appropriate treatment.

Methods: We conducted a retrospective observational study of 17 patients diagnosed with CTD-ILD between July 2024 and July 2025 at a tertiary care center in Central India. Data collected included demographic characteristics, connective tissue disease subtype, high-resolution CT (HRCT) pattern, autoantibody profile, spirometry findings, prior exposure to anti-tubercular therapy, and treatment received.

Results: The mean age was 52.7 years, with female predominance. Connective tissue disease subtypes included Sjögren syndrome (4/17, 24%), systemic lupus erythematosus (3/17, 18%), inflammatory myositis (3/17, 18%), systemic sclerosis (2/17, 12%), mixed connective tissue disease (3/17, 18%), and overlap syndromes (2/17, 12%). The HRCT patterns showed nonspecific interstitial pneumonia (NSIP) in 11/17 (65%), usual interstitial pneumonia (UIP) in 5/17 (29%), and lymphocytic interstitial pneumonia (LIP) in 1/17 (6%). All patients were positive for antinuclear antibodies, with disease-specific extractable nuclear antigen antibodies including Ro52, Jo-1, and Scl-70. Nine patients (9/17, 53%) had received empirical anti-tubercular therapy prior to diagnosis. Spirometry was available in 7/17 patients (41%); the median forced vital capacity was 75% of predicted, and the mean forced expiratory volume in one second to forced vital capacity ratio was 66%. Treatment included mycophenolate mofetil (9/17, 53%) and nintedanib (7/17, 41%), and one patient each received cyclophosphamide and rituximab, respectively.

Conclusions: In this central Indian cohort, CTD-ILD most commonly presented with an NSIP pattern and was frequently misdiagnosed as tuberculosis, resulting in exposure to empirical anti-tubercular therapy. Early evaluation with HRCT and autoimmune serological testing is essential to ensure accurate diagnosis and timely initiation of appropriate immunosuppressive and antifibrotic therapy.

## Introduction

Connective tissue disease-associated interstitial lung disease (CTD-ILD) is a major cause of morbidity among patients with autoimmune disorders and represents a significant subset of diffuse parenchymal lung diseases worldwide [1,2]. Registry-based data from India suggest that CTD-ILD accounts for nearly 13% to 20% of interstitial lung disease cases in tertiary care settings [3]. In tuberculosis-endemic regions such as India, patients presenting with chronic respiratory symptoms and diffuse radiological infiltrates are frequently presumed to have pulmonary tuberculosis, resulting in empirical initiation of anti-tubercular therapy and delayed diagnosis of CTD-ILD [3,4].

Interstitial lung disease occurs in approximately 15% to 30% of patients with connective tissue disorders, with higher prevalence in systemic sclerosis and inflammatory myositis, and contributes substantially to disease-related mortality [1]. High-resolution CT (HRCT) plays a central role in identifying interstitial lung disease patterns, including nonspecific interstitial pneumonia (NSIP), usual interstitial pneumonia (UIP), and lymphocytic interstitial pneumonia (LIP) [5]. Serological profiling using antinuclear antibody (ANA) and extractable nuclear antigen (ENA) testing helps establish the underlying connective tissue disease and guide treatment [6].

Previous Indian studies have demonstrated frequent misdiagnosis of interstitial lung disease as tuberculosis, leading to unnecessary anti-tubercular therapy exposure and delayed immunosuppressive therapy [7-9]. Early multidisciplinary evaluation involving pulmonologists, rheumatologists, and radiologists is essential for accurate diagnosis and management [10-12]. Evidence from prior literature further highlights the clinical spectrum and outcomes of CTD-ILD [13]. This study aimed to evaluate the clinical profile, radiological patterns, serological characteristics, and frequency of misdiagnosis as tuberculosis among patients with CTD-ILD at a tertiary care center in Central India.

## Materials and methods

This retrospective observational study was conducted at the LN Medical College and Research Center and JK Hospital in Bhopal, Madhya Pradesh, India, a tertiary care referral center in central India, and was approved by the same (approval no. LNMC&RC/Dean/2023/Ethics/278). The study included patients diagnosed with CTD-ILD between July 2024 and July 2025.

Eligible patients were identified through hospital medical records. Adult patients with a confirmed diagnosis of connective tissue disease and radiological evidence of interstitial lung disease on HRCT were included. Diagnosis of connective tissue disease was established based on clinical evaluation supported by positive autoimmune serology, including ANA and/or ENA antibody testing. Inclusion criteria comprised adult patients (≥18 years) diagnosed with connective tissue disease with HRCT evidence of interstitial lung disease and positive autoimmune serology (ANA and/or ENA antibodies). Exclusion criteria included patients with microbiologically confirmed active pulmonary tuberculosis, infective causes of interstitial lung disease, and cases with incomplete or insufficient medical records for analysis.

Demographic and clinical data were retrieved retrospectively from electronic and physical medical records. Collected variables include age, sex, connective tissue disease subtype, HRCT pattern, autoimmune serological profile, spirometry findings where available, prior exposure to anti-tubercular therapy (ATT), and treatment received following diagnosis. The HRCT findings were reviewed in the radiology reports and categorized as NSIP, UIP, LIP, or other interstitial lung disease patterns. Pulmonary function test results were recorded when available and included forced vital capacity (FVC) and forced expiratory volume (FEV) in one second to FVC ratio (FEV/FVC).

Descriptive statistical analysis was performed using SPSS Statistics version 25.0 (IBM Corp., Armonk, NY, USA). Continuous variables were expressed as mean ± standard deviation or median values where appropriate, while categorical variables were summarized as frequencies and percentages.

## Results

**Figure 1:**
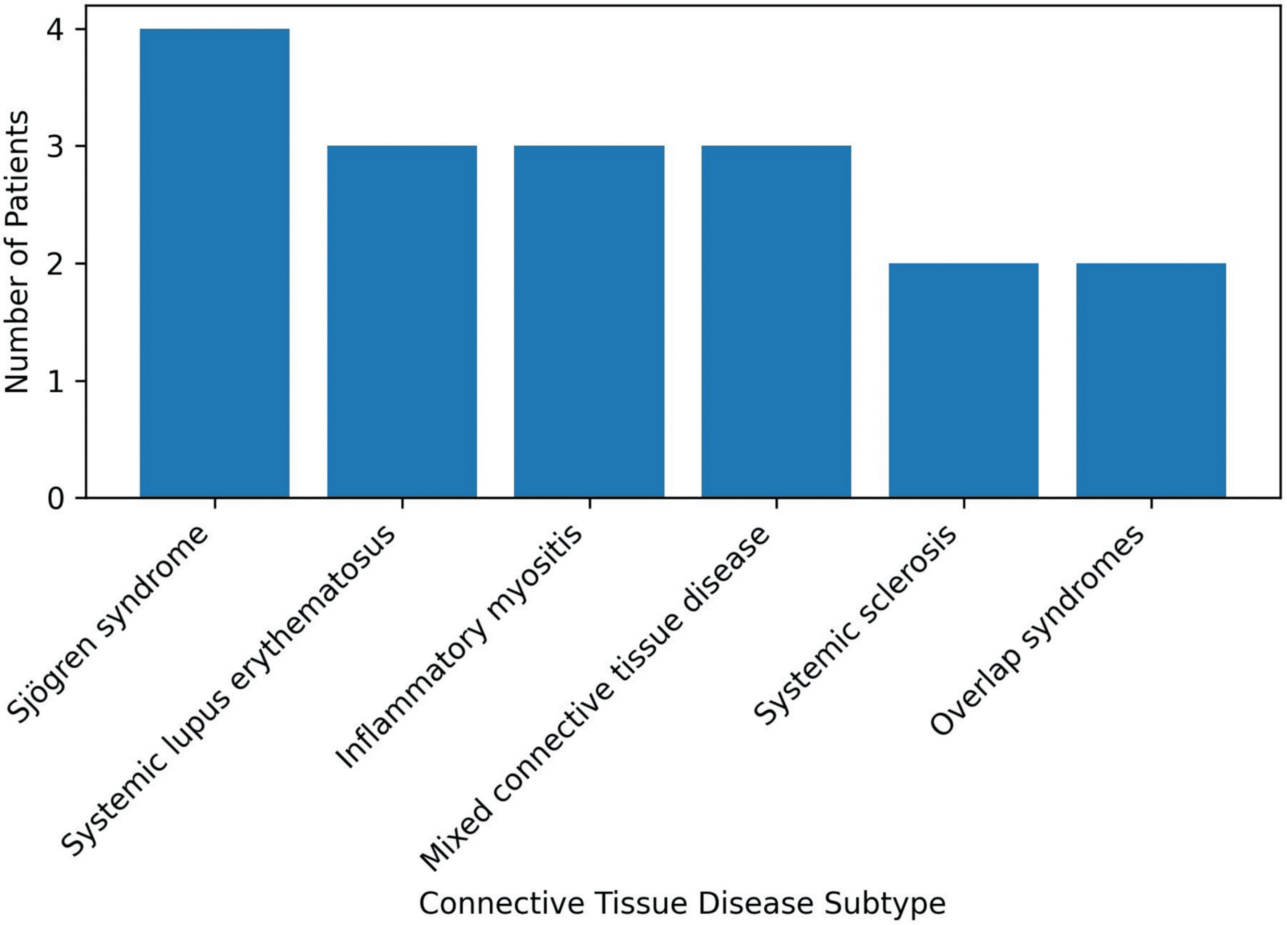
Distribution of connective tissue disease subtypes among patients with CTD-ILD The bar graph illustrates the frequency of connective tissue disease subtypes, with Sjögren syndrome being the most common subtype, followed by systemic lupus erythematosus, inflammatory myositis, mixed connective tissue disease, systemic sclerosis, and overlap syndromes. CTD-ILD: Connective tissue disease-associated interstitial lung disease Source: Institutional data from LN Medical College and JK Hospital (Bhopal, MP, IND)

A total of 17 patients with CTD-ILD were included in the study. The mean age was 52.7 ± 11.4 years, with a female predominance (11/17, 65%). The distribution of underlying connective tissue disease subtypes showed that Sjögren syndrome was the most common (4/17, 24%), followed by systemic lupus erythematosus (3/17, 18%), inflammatory myositis (3/17, 18%), mixed connective tissue disease (3/17, 18%), systemic sclerosis (2/17, 12%), and overlap syndromes (2/17, 12%). The distribution of connective tissue disease subtypes is illustrated in Figure 1.

**Figure 2:**
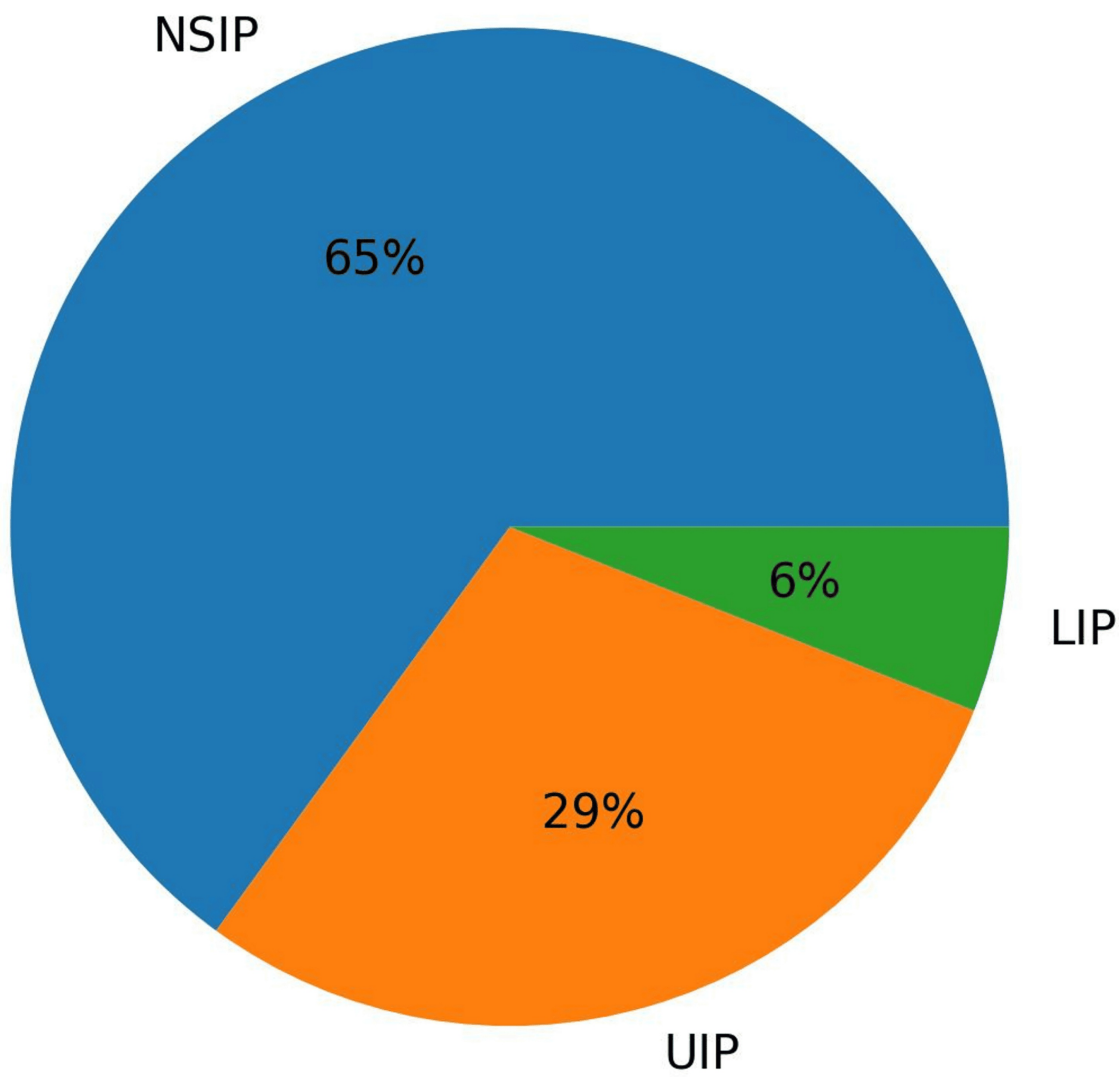
Distribution of HRCT patterns in CTD-ILD The pie chart shows the distribution of HRCT patterns among the study population (total n = 17); NSIP was the most common pattern (65%), followed by UIP (29%) and LIP (6%). HRCT:  High-resolution computed tomography, CTD-ILD: Connective tissue disease–associated interstitial lung disease, NSIP: Nonspecific interstitial pneumonia, UIP: Usual interstitial pneumonia, LIP: Lymphocytic interstitial pneumonia Source: Institutional data from LN Medical College and JK Hospital (Bhopal, MP, IND)

The HRCT findings demonstrated that NSIP was the predominant radiological pattern (11/17, 65%), followed by UIP (5/17, 29%) and LIP (1/17, 6%). The distribution of HRCT patterns among patients is shown in Figure 2. All patients (17/17, 100%) were positive for ANA. Frequently detected ENA antibodies included Ro52, Jo-1, and Scl-70. A total of 9/17 patients (53%) had received empirical anti-tubercular therapy before the correct diagnosis of CTD-ILD.

Spirometry was available in 7/17 patients (41%). The median FVC was 75% of predicted, and the mean FEV/FVC ratio was 66%, consistent with a predominantly restrictive ventilatory pattern. Regarding treatment, mycophenolate mofetil was administered in 9/17 patients (53%), nintedanib in 7/17 (41%), and one patient each (1/17, 6%) received cyclophosphamide and rituximab, respectively.

## Discussion

**Figure 3:**
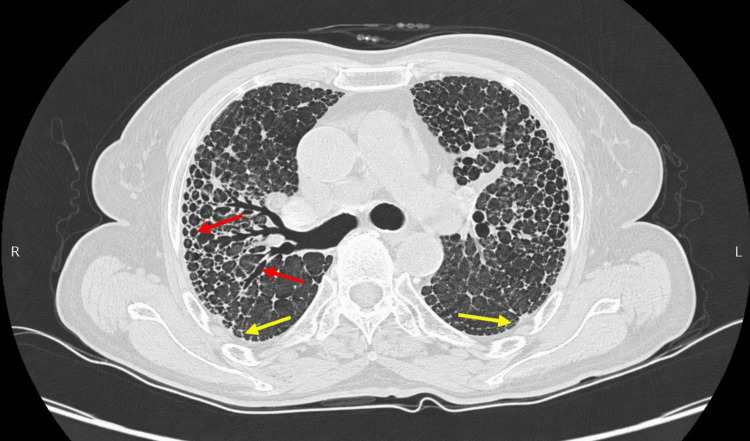
Axial HRCT of the chest showing fibrotic interstitial lung disease pattern in a patient with CTD-ILD Yellow arrows point to bilateral, symmetrical, basal-predominant reticular opacities with subpleural distribution. Red arrows show the areas of honeycombing and traction bronchiectasis, consistent with a fibrotic interstitial lung disease pattern. The patient was initially treated with empirical ATT at a peripheral center based on radiological suspicion of pulmonary tuberculosis. Subsequent clinical evaluation, autoimmune serology, and multidisciplinary review confirmed CTD-ILD. HRCT: High-resolution computed tomography, CTD-ILD: Connective tissue disease–associated interstitial lung disease, ATT: Anti-tubercular therapy Source: Institutional data from LN Medical College and JK Hospital (Bhopal, MP, IND)

Connective tissue disease-associated interstitial lung disease represents a clinically significant subset of diffuse parenchymal lung diseases and is an important contributor to morbidity in patients with autoimmune disorders. Recent global studies have further described the evolving spectrum of fibrotic interstitial lung diseases and CTD-ILD patterns across different populations [13,14]. The HRCT plays a central role in the classification and diagnostic evaluation of interstitial lung disease patterns, allowing differentiation of NSIP, UIP, and other radiological subtypes [1,5]. In the present study, NSIP was the predominant HRCT pattern (65%), consistent with previous international and Indian cohorts that have reported NSIP as the most frequent radiological manifestation in CTD-ILD [4,9]. Representative HRCT imaging demonstrating fibrotic interstitial lung disease features is shown in Figure 3.

Serological profiling, including ANA and ENA testing, is essential for identifying the underlying connective tissue disorder and guiding therapy [6]. In our cohort, all patients were ANA-positive, reinforcing the importance of autoimmune screening in patients presenting with diffuse interstitial lung disease. Frequently detected ENA antibodies such as Ro52, Jo-1, and Scl-70 further supported subtype classification and informed management decisions.

India bears a high burden of tuberculosis, and radiological overlap between CTD-ILD and pulmonary tuberculosis often leads to diagnostic uncertainty. Previous Indian studies have highlighted frequent misdiagnosis of interstitial lung disease as tuberculosis, resulting in unnecessary exposure to ATT and delayed immunosuppressive treatment [7,8]. In our study, 53% of patients had received empirical ATT prior to definitive diagnosis, underscoring the clinical challenge in tuberculosis-endemic regions. These findings align with registry-based observations demonstrating variability in diagnostic practices and delayed referral patterns in ILD management across India [3,9].

The implications of delayed diagnosis are clinically significant, as untreated CTD-ILD may progress to irreversible fibrosis. Early identification through HRCT, combined with serological testing and multidisciplinary discussion involving pulmonologists, rheumatologists, and radiologists, is crucial for accurate diagnosis and timely initiation of immunosuppressive or antifibrotic therapy [2,8]. Our findings emphasize the need for structured diagnostic pathways to reduce inappropriate ATT exposure and improve patient outcomes. Although limited by sample size, this study contributes to regional data on CTD-ILD patterns and highlights the ongoing issue of tuberculosis misdiagnosis in autoimmune-related interstitial lung disease.

## Conclusions

Connective tissue disease-associated interstitial lung disease represents an important and often underrecognized cause of diffuse parenchymal lung disease in tuberculosis-endemic regions. Overlapping clinical and radiological features with pulmonary tuberculosis may lead to diagnostic uncertainty and inappropriate empirical therapy, resulting in delays in initiating disease-specific management. Early evaluation using HRCT combined with autoimmune serological testing and multidisciplinary assessment is essential for accurate diagnosis. Strengthening awareness of CTD-ILD and implementing structured diagnostic pathways may help reduce misdiagnosis, avoid unnecessary ATT exposure, and improve clinical outcomes in patients presenting with chronic interstitial lung disease.
